# The anatomical bone corridor study of schanz screw placement in adolescent pelvic ring fractures via three-dimensional CT

**DOI:** 10.3389/fsurg.2026.1816272

**Published:** 2026-05-01

**Authors:** Xiang Ren, Fang Xie, Jie Hu

**Affiliations:** 1 Department of Orthopedics, School of Medicine, Chengdu Women's and Children’s Central Hospital, University of Electronic Science and Technology of China, Chengdu, Sichuan Province, China; 2 Department of Ultrasound, Sichuan Academy of Medical Sciences and Sichuan Provincial People’s Hospital, Affiliated Hospital of University of Electronic Science and Technology of China, Chengdu, Sichuan Province, China

**Keywords:** adolescents, anatomical channels, computed tomography (CT), external fixation, pelvic fractures

## Abstract

**Purpose:**

To define the anatomical boundaries of potential safe corridors for the placement of Schanz screws during external fixation of unstable pelvic fractures in normally developed adolescents aged 11–14 years.

**Patients and methods:**

Pelvic CT scans of 34 patients (18 males, 16 females; mean age 12 years) were reconstructed into 3D models using Mimics 21.0. A supine coordinate system was defined using anatomical landmarks, including the anterior superior iliac spine (ASIS), posterior superior iliac spine (PSIS), and anterior inferior iliac spine (AIIS). Cross-sectional measurements of the iliac wing and supra-acetabular corridors were performed, simulating 4 mm Schanz screw trajectories and documenting entry points, angles, and corridor dimensions.

**Results:**

Iliac Wing Corridor: The morphology transitioned from proximal rectangular to mid-corridor dumbbell-shaped, with distal funnel-like narrowing. Anterior/posterior corridor lengths measured 54.2–58.6 mm and 68.1–73.1 mm (95% CI), respectively. Screw I1 entry (16.9–18.7 mm posterior to ASIS; 95% CI) required horizontal/sagittal angles of 22.5–25.8° and 53.7–61.2° (95% CI). Screw I2 entry (49.4–53.6 mm posterior to ASIS; 95% CI) necessitated horizontal/sagittal angles of 21.5–25.1° and 61.3–66.1° (95% CI).

**Supra-acetabular corridor:**

S-shaped with mid-corridor constriction. The longest trajectory (116.7–125.3 mm; 95% CI) aligned with the AIIS-PSIS central plane, while the shortest (95.9–105.9 mm; 95% CI) occurred in the periacetabular region. Screw A insertion via the AIIS-PSIS plane required horizontal/sagittal angles of 58.2–61.3° and 23.0–25.6° (95% CI), respectively.

**Conclusion:**

Both iliac wing and supra-acetabular corridors demonstrated safe accommodation of 4 mm Schanz screws in this age group. Adherence to predefined entry points and angles is critical to minimize perforation risk and optimize fixation stability. Patient-specific computer-assisted preoperative surgical planning is particularly well-suited for adolescents in enhancing surgical safety.

## 1 Introduction

Unstable pelvic fractures in adolescents represent high-energy traumatic injuries that are accompanied by substantial morbidity and mortality (1). Surgical treatment is frequently required to restore the integrity and stability of the pelvic ring structure. This is not only crucial for effective trauma management but also of key significance for early functional rehabilitation, the reduction of complications, and the improvement of prognosis.

Owing to the intricate anatomical structure of the pelvis, minimally invasive treatment for pelvic fractures remains one of the formidable challenges in orthopedic trauma surgery. The pelvis is a ring-shaped structure composed of the innominate bone, which encompasses the paired iliac, pubic, and ischial bones, along with the central sacrum and coccyx. Multiple joints are formed between these bones, with a plethora of vital blood vessels and nerves traversing the anatomical cavities (2). It serves as a conduit for transmitting mechanical loads between the spine and lower limbs through the hip joint, while the pelvic cavity it encloses provides shelter and protection for internal organs, blood vessels, and nerves. Research has demonstrated that in adults, there exist well-defined bone anatomical corridors within the pelvic ring that allow for the precise percutaneous placement of internal (or external) fixation screws (3–5), thereby facilitating the reduction and stabilization of fractures through minimally invasive surgical procedures (6, 7). However, in pediatric patients, the presence of growth plates, the smaller three-dimensional dimensions of the pelvis, and the marked variations in pelvic shape and size across different developmental stages have collectively contributed to a dearth of research on bone anatomical corridors in the pediatric pelvis thus far. This has, in turn, limited the widespread application of minimally invasive techniques in the management of pediatric pelvic fractures.

External fixation, as one of the commonly employed minimally invasive fixation methods for treating pelvic fractures, can serve as a definitive treatment modality and a temporary stabilizing intervention for polytrauma patients (8–10). When combined with posterior pelvic ring fixation, this technique achieves biomechanically stable reduction of unstable pelvic fractures (11) and has demonstrated successful application in the management of pediatric pelvic fractures (12). However, given the inherent anatomical and developmental disparities between pediatric and adult populations (13), it has been observed that conventional fluoroscopy-guided techniques for screw placement carry several risks in children, including cortical breach, physeal injury, unstable fixation, and increased radiation exposure (7, 12).

This study employed a high—resolution pelvic computed tomography (CT) dataset, which was derived from a cohort of normally developed adolescents aged 11 to 14 years. Three—dimensional modeling, computer—assisted preoperative planning, and visual analysis of osseous corridors were employed to investigate whether anatomically suitable bone channels for Schanz screw placement exist in this age group, and their boundaries were defined. The primary objective was to optimize surgical safety parameters and enhance clinical outcomes.

## 2 Methods

This retrospective cohort study was conducted in compliance with the Declaration of Helsinki and received ethical approval from the study institution (IRB No. 2023-102). Written informed consent was waived per institutional guidelines due to the retrospective nature of data collection.

### 2.1 Data collection

Pelvic CT scans (*n* = 34) performed between January 2020 and December 2024 were retrieved from the institutional Picture Archiving and Communication System (PACS). All examinations were acquired using a Siemens SOMATOM Force 64-detector row scanner operating under standardized low-dose protocols with the following parameters: collimation width 0.625 mm, reconstruction increment 0.3 mm, tube voltage 120 kVp, and automated tube current modulation (80–250 mA).

### 2.2 Eligibility criteria

Patients were included if they met the following criteria at the time of imaging: aged 11–14 years, and CT scans performed for clinical indications unrelated to pelvic pathology. Exclusion criteria comprised: history of pelvic fractures; active infections, neoplasms, or metabolic bone disease; prior pelvic surgical intervention; or incomplete imaging datasets precluding comprehensive analysis.

### 2.3 Three-Dimensional (3D) reconstruction, anatomical landmark localization and coordinate system establishment

Step 1: 3D reconstruction.

**Figure 1:**
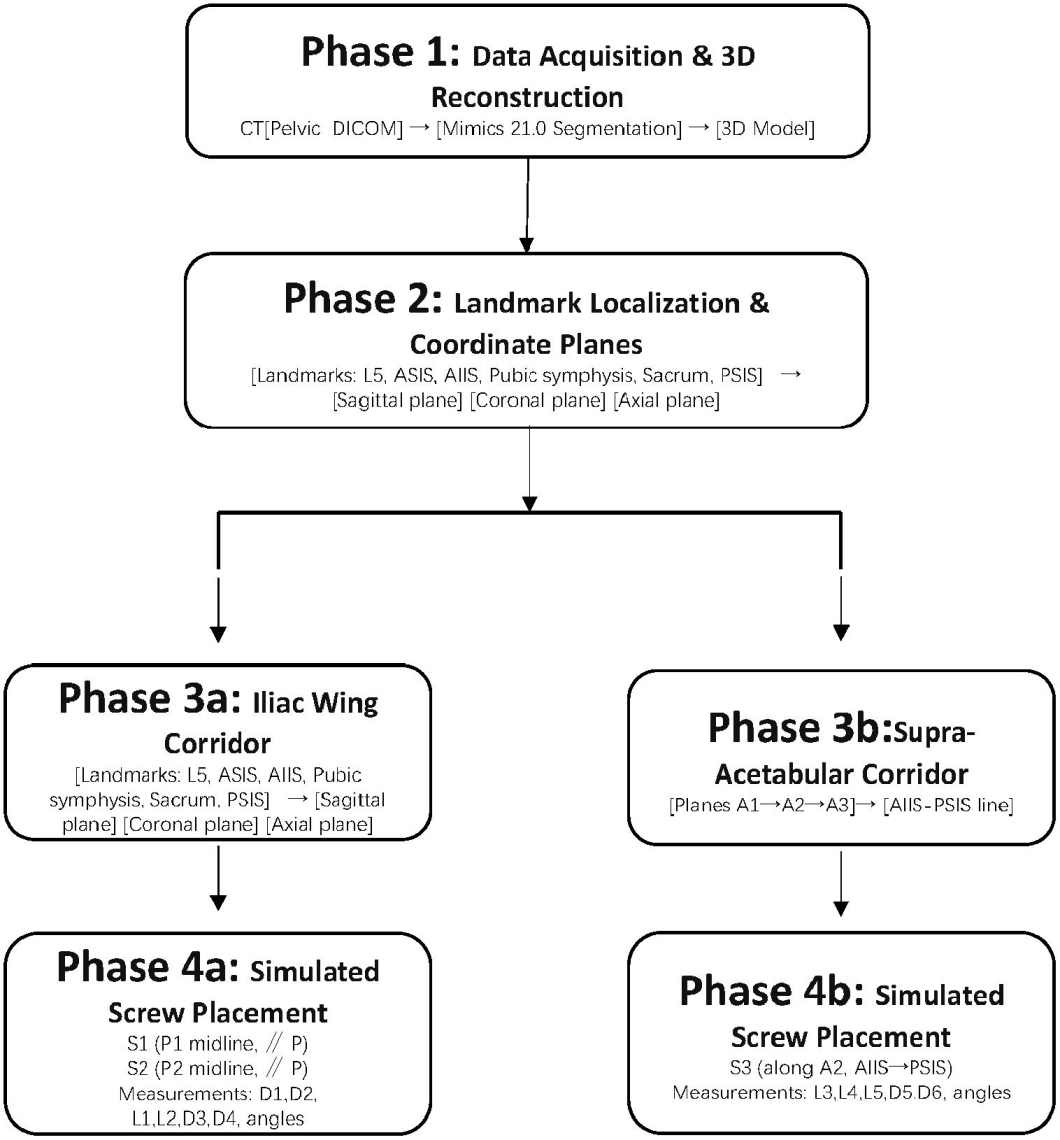
Schematic workflow of the 3D anatomical analysis and simulated screw placement. The workflow consists of four phases. Phase 1: Pelvic CT DICOM data were imported into Mimics 21.0 for segmentation and 3D reconstruction. Phase 2: Anatomical landmarks (L5, ASIS, AIIS, pubic symphysis, sacral promontory, PSIS) were identified, and the sagittal, coronal, and axial planes were defined. Phase 3: Two bone corridors were defined—(a) the iliac wing corridor via sequential posterior translation of reference plane *P* to P1 and P2, identifying points I1 and I2; (b) the supra-acetabular corridor via planes A1, A2, and A3 based on the AIIS and PSIS landmarks. Phase 4: Simulated placement of 4 mm Schanz screws (S1, S2 in the iliac wing corridor; S3 in the supra-acetabular corridor) and the corresponding measurements. 3D, three-dimensional; AIIS, anterior inferior iliac spine; ASIS, anterior superior iliac spine; CT, computed tomography; L5, fifth lumbar vertebra; PSIS, posterior superior iliac spine.

DICOM files of pelvic CT scans were imported into Materialise MIMICS Innovation Suite Medical (v21.0; Materialise NV, Leuven, Belgium) to perform image segmentation and 3D reconstruction of the pelvis and lumbosacral spine. Figure 1 provides a schematic overview of the entire workflow from 3D reconstruction to simulated screw placement.

Step 2: Landmark localization.

The following anatomical landmarks were identified on the 3D pelvic model:

Midpoint of the anterior margin of the inferior endplate of the fifth lumbar vertebra (L5). Most anterior points of the anterior superior iliac spine (ASIS) and anterior inferior iliac spine (AIIS), with contour line demarcation.

Transition zone between the ASIS and AIIS along the anterior iliac crest.

Midpoint of the pubic symphysis.

Midpoint of the anterior margin of the sacral promontory.

Posterior superior iliac spine (PSIS) contour line and the midpoint of its maximum transverse diameter.

Step 3: Coordinate plane definition.

**Figure 2:**
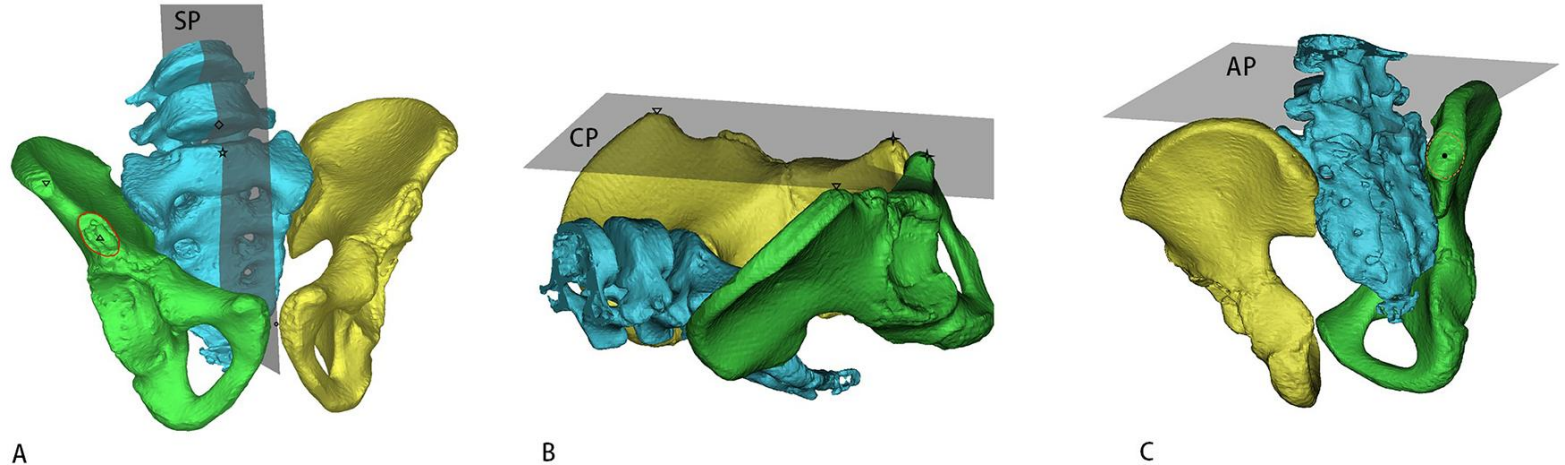
Three-Dimensional pelvic model with landmark localization and coordinate system. This figure shows the three-dimensional anatomical localization of key pelvic landmarks and the establishment of a standardized coordinate system. **(A)** shows the sagittal plane, **(B)** shows the coronal plane, and **(C)** shows the axial plane. Annotations: Anterior margin midpoint of the inferior endplate of the fifth lumbar vertebra (◇), Anterior midpoint of the sacral promontory (⋆), Anterior-most point of the anterior superior iliac spine (ASIS; ▽), Anterior-most point of anterior inferior iliac spine (AIIS; △) with its contour delineated by a red solid line. Anterior pubic tubercles (✧), Midpoint of the pubic symphysis (○). Posterior superior iliac spine (PSIS), outlined by a red dashed circle, with its maximum transverse diameter midpoint (●). The coordinate system uses sagittal (SP), coronal (CP), and axial (AP) planes.

Coordinate planes were defined using anatomical landmarks as follows (2):

Sagittal plane: Passed through the midpoint of the L5 inferior endplate anterior margin, the midpoint of the sacral promontory anterior margin, and the midpoint of the pubic symphysis (Figure 2A);

Coronal plane: Extended through the bilateral ASIS and anterior pubic tubercles (Figure 2B).

Axial plane: Oriented perpendicular to both the sagittal and coronal planes (Figure 2C).

### 2.4 Anatomical corridor definition

Anatomical pathways were delineated through systematic construction of virtual cut planes. Two corridors were defined: the iliac wing corridor and the supra-acetabular corridor.

#### 2.4.1 Iliac crest corridor identification

Step 1: Reference plane P. A reference plane (P) was constructed tangent to the most anterior point of the ASIS-AIIS transition zone, oriented perpendicular to the medial and lateral surfaces of the iliac wing.

**Figure 3:**
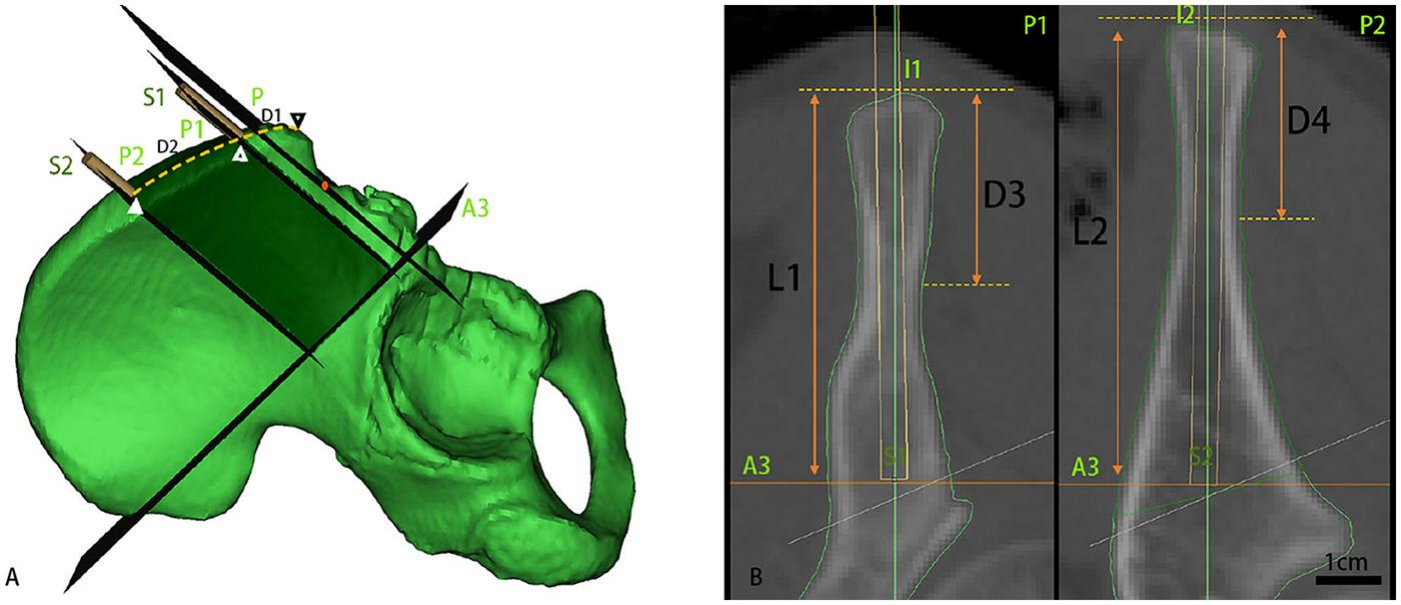
Iliac crest corridor. This figure shows the Iliac Crest Corridor, marked as a green zone in pelvic model. In **(A)**, Key annotations: Anterior-most point of the anterior superior iliac spine (ASIS; ▽). I1: The entry point for the screw at the most anterior part of the iliac crest corridor, represented by an open white triangle. I2: The entry point for the screw at the most posterior part of the iliac crest corridor, represented by a solid white triangle. Most anterior point of the ASIS-AIIS transition zone: red solid ellipse. **(B)** presents the measurements within the Iliac Crest Corridor. P: the reference plane; P1: the most anterior plane of the corridor; P2: the most posterior plane of the corridor; A3: the Plane passes through the distal endpoints of both the AIIS and PSIS contour lines; D1: the distance between point I1 and the most anterior point of the anterior superior iliac spine; D2: the distance between point I2 and the most anterior point of the anterior superior iliac spine; D3: the distance from the narrowest part of the medial and lateral cortices of the bone in the corridor at plane P1 to point I1; D4: the distance from the narrowest part of the medial and lateral cortices of the bone in the corridor at plane P2 to point I2; S1: the anterior Schanz screw; S2: the posterior Schanz screw; L1: the length of the anterior corridor; L2: the length of the posterior corridor.

Step 2: Posterior translation. Plane *P* was translated posteriorly along the iliac crest while maintaining parallelism. During translation, the iliac wing was monitored for minimal wing thickness: when the narrowest distance between medial and lateral iliac wing cortical reached 4 mm (compatible with 4 mm Schanz screw placement), the midline intersection point on the superior iliac crest surface was labeled I1, and the corresponding section designated P1. Subsequent posterior translation of plane *P* identified a second 4 mm thickness threshold, with the midline intersection point labeled I2 and section designated P2. The region between P1 and P2 constituted the iliac crest corridor as demonstrated in Figure 3A.

#### 2.4.2 Supra-Acetabular corridor planning

Step 1: Boundary planes (A1 and A3). Two primary virtual cut planes were established perpendicular to the iliac wing surface: Plane A1 passes through the proximal endpoints of both the AIIS and PSIS contour lines, while Plane A3 is defined by their distal endpoints. The region between these planes forms the supra-acetabular corridor.

**Figure 4:**
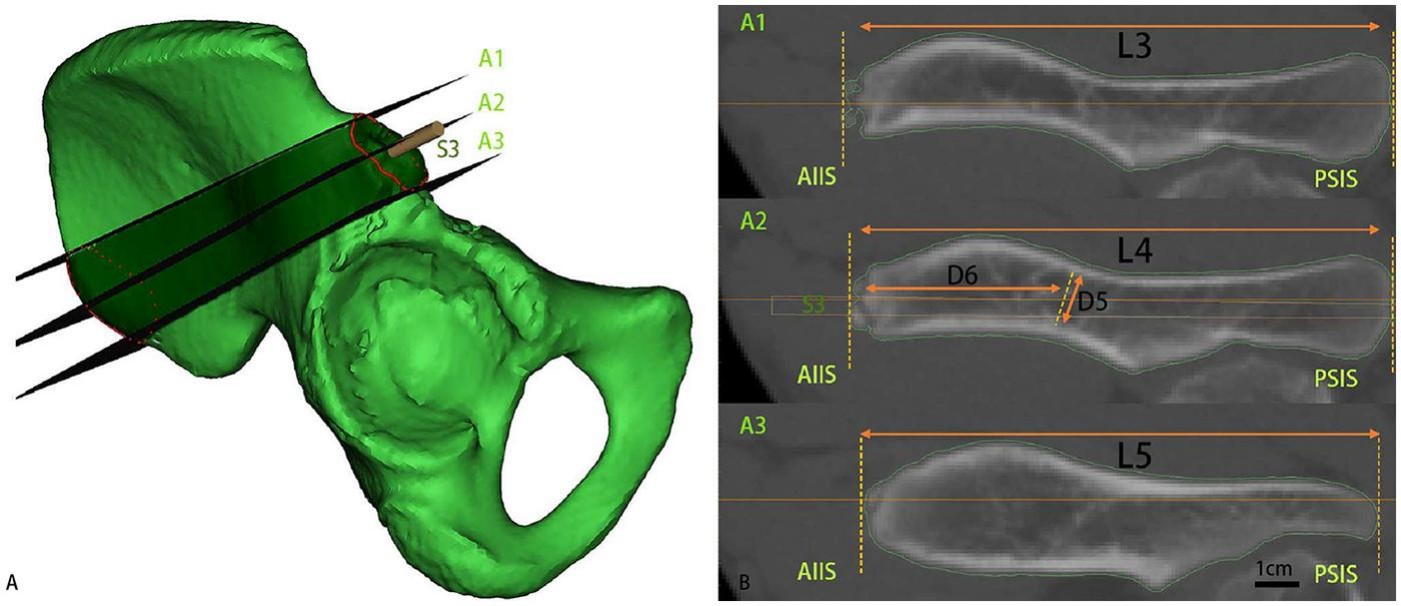
Supra-Acetabular corridor. This figure shows the Supra-Acetabular Corridor, marked as a green zone in pelvic model. In **(A)**, Key annotations: Anterior Inferior Iliac Spine (AIIS): Red solid line. Posterior Superior Iliac Spine (PSIS): Red dashed circle. **(B)** presents measurements in the Supra-Acetabular Corridor. A1: the Plane passes through the proximal endpoints of both the AIIS and PSIS contour lines; A2: the Plane passes through the most anterior point of the AIIS and the midpoint of the maximum transverse diameter of the PSIS contour; A3: the Plane passes through the distal endpoints of both the AIIS and PSIS contour lines; L3: Axial lengths of sections A1; L4: Axial lengths of sections A2; L5: Axial lengths of sections A3; D5: the medial and lateral cortical spacing of the iliac bone at the mid-corridor constriction of section A2; D6: the distance from the most anterior point of the anterior inferior iliac spine to the mid-corridor constriction.

Step 2: Mid-corridor plane (A2). An additional perpendicular plane (A2) is constructed through two critical landmarks—the most anterior point of the AIIS and the midpoint of the maximum transverse diameter of the PSIS contour as demonstrated in Figure 4A.

### 2.5 Simulated screw placement and measurement protocol

#### 2.5.1 Simulated screw placement

Considering the smaller pelvic dimensions in adolescents, 4 mm Schanz screws were selected for simulated insertion through the midline of sections P1 and P2, following a trajectory parallel to reference plane P, and were designated as S1 and S2 screws as shown in Figure 3A. Notably, this selection aligns with clinical practice (12), as these screws are commonly used in smaller pelvic anatomies and have been shown to provide adequate fixation stability in pediatric populations. Subsequently, a 4 mm Schanz screw S3 was placed through plane A2, aligned with the line connecting the most anterior point of the AIIS to the midpoint of the PSIS contour's maximum transverse diameter as shown in Figure 4B.

#### 2.5.2 Measurements

All measurements were conducted using a calibrated reference framework to ensure reproducibility. The following parameters were recorded:

#### 2.5.3 Measurements for the iliac wing corridor

D1, D2: Distance from the most anterior point of the ASIS to I1 (D1) and to I2 (D2).

L1, L2: Vertical distance from sections P1 (through I1) and P2 (through I2) to the inferior acetabular pathway plane (A3)—representing iliac wing corridor lengths.

D3, D4: Medial-lateral cortical spacing at points I1 (D3) and I2 (D4) (Figure 3B).

**Figure 5:**
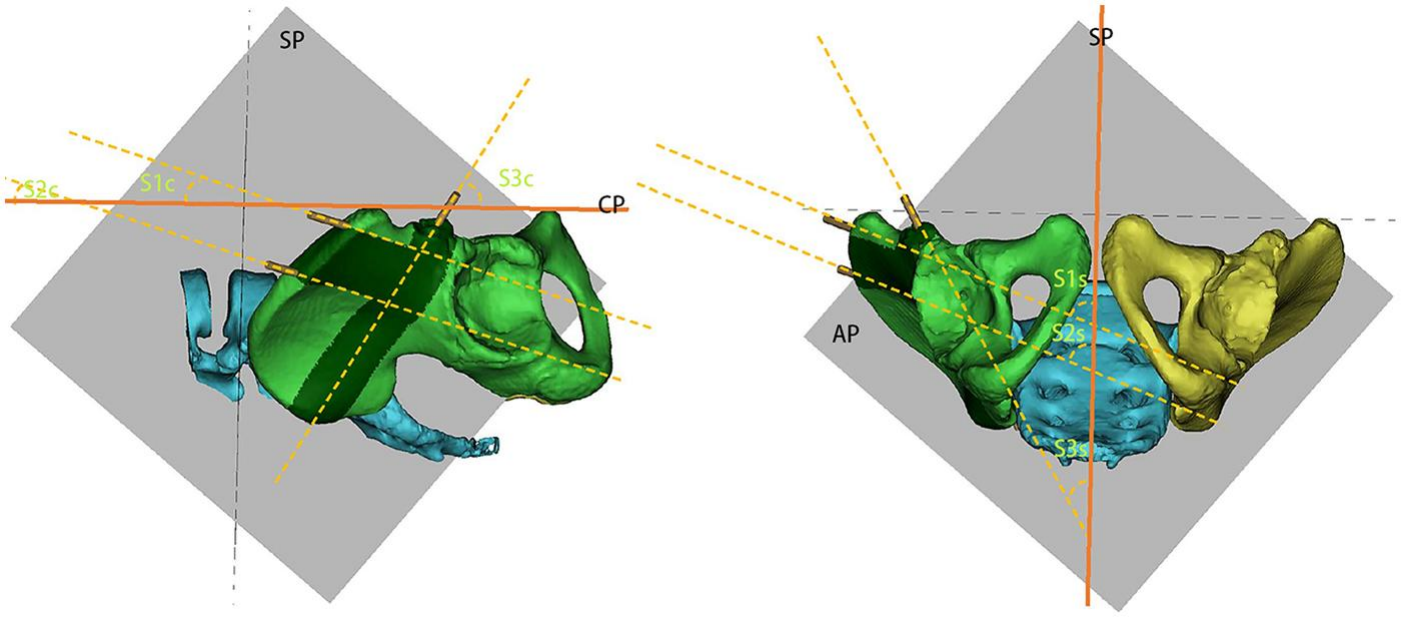
Angular measurement for schanz screws. This figure depicts the angles between the Schanz screws (S1, S2, S3) and their projections on the sagittal (SP) or coronal planes (CP) were recorded as Sagittal1 (S1s), Coronal1 (S1c), Sagittal2 (S2s), Coronal2 (S2c), Sagittal3 (S3s), and Coronal3 (S3c).

Angles: Sagittal1 (S1s), Coronal1 (S1c), Sagittal2 (S2s), Coronal2 (S2c)—the angles between the Schanz screws (S1, S2) and their projections on the sagittal or coronal planes (Figure 5).

Measurements for the supra-acetabular corridor

L3, L4, L5: Axial lengths of sections A1, A2, and A3.

D5: Medial-lateral cortical spacing at the mid-corridor constriction of section A2.

D6: Distance from the most anterior point of the AIIS to the mid-corridor constriction (Figure 4B).

Angles: Sagittal3 (S3s), Coronal3 (S3c)—the angles between screw S3 and its projections on the sagittal or coronal planes (Figure 5).

### 2.6 Sample size justification

A formal *a priori* power analysis was not performed due to the exploratory nature of this study and the absence of prior data in adolescents aged 11–14 years. The sample of 34 subjects represents all eligible consecutive cases from a five-year period at a single center, which is a realistic cohort given that pelvic CT is not routinely performed in children. The narrow 95% confidence intervals for all primary parameters support the precision of our estimates.

### 2.7 Statistical methods

Analyses used GraphPad Prism 10 and R 4.2. Continuous data were normality-tested (Shapiro–Wilk, *p* > 0.05) and presented as mean ± SD with 95% CI. Interobserver reliability was assessed with ICC {two-way random, absolute agreement, single measurement [ICC(2,1)]} using R's icc(); reliability followed Koo & Li (2016): <0.50 = poor, 0.50–0.74 = moderate, 0.75–0.89 = good, ≥0.90 = excellent. Sex comparisons (height and weight) used two independent *t*-tests (*α* = 0.05, two-tailed); no multiple comparison correction was needed. No formal power analysis was performed (exploratory descriptive study); precision is supported by narrow 95% CIs (mean width: 5.7 mm for lengths, 4.2° for angles). This limitation is acknowledged in the Discussion.

## 3 Results

A total of 34 eligible patients (18 males, 16 females; mean age 12.3 ± 0.8 years) were enrolled in the study. The height and weight of included patients are shown in Table 1. Two radiologists independently performed anatomical landmark localization and subsequent parameter measurements. Interobserver reliability demonstrated excellent agreement across all parameters, with intraclass correlation coefficients (ICC) ranging from 0.93 to 0.98.

**Table 1:** Comprehensive anatomical measurements of iliac and acetabular corridors in adolescent pelvic ring (*n* = 34).

Parameter	Mean ± SD	95% CI	Unit
D1	17.8 ± 2.7	16.9–18.7	mm
D2	51.5 ± 6.0	49.4–53.6	mm
L1	56.4 ± 6.2	54.2–58.6	mm
L2	70.6 ± 7.2	68.1–73.1	mm
D3	27.6 ± 4.0	26.2–29.0	mm
D4	32.7 ± 5.1	30.9–34.4	mm
L3	118.1 ± 11.3	114.2–122.0	mm
L4	121.0 ± 12.4	116.7–125.3	mm
L5	100.9 ± 14.4	95.9–105.9	mm
D5	12.9 ± 1.7	12.3–13.5	mm
D6	49.7 ± 5.3	47.8–51.5	mm
S1c	24.2 ± 4.8	22.5–25.8	
S2c	23.3 ± 5.2	21.5–25.1	
S1s	57.5 ± 10.8	53.7–61.2	
S2s	63.7 ± 7.0	61.3–66.1	
S3c	59.8 ± 4.4	58.2–61.3	
S3s	24.3 ± 3.7	23.0–25.6	

Data presented as mean ± standard deviation (SD) with 95% confidence interval (CI), Angular measurements in degrees (°), linear measurements in millimeters (mm).

The iliac wing corridor, running along the anterior iliac crest, exhibits a proximal-to-distal morphological transformation, starting as a rectangular cross-section, transitioning to a dumbbell-shaped mid-corridor, and culminating in a funnel-like expansion near the supra-acetabular region. Its anatomical constraints include superior narrowing, inferior widening, lateral convexity, medial concavity, and restricted anterior-posterior dimensions. The study simulated parallel placement of two 4.0 mm Schanz screws with the following parameters: The anterior entry point I1 was positioned 16.9–18.7 mm (95% CI) from the ASIS, while the posterior entry point I2 was located 49.4–53.6 mm (95% CI) from the ASIS, yielding a mean inter-screw distance of 35 mm. The anterior and posterior corridor lengths averaged 54.2–58.6 mm (95% CI) and 68.1–73.1 mm (95% CI), respectively. A critical mid-corridor constriction located around 30 mm deep to the iliac crest surface. Angulation analysis revealed Schanz S1 oriented at 22.5–25.8° (95% CI) relative to the horizontal plane and 53.7–61.2° (95% CI) relative to the sagittal plane, while Schanz S2 showed horizontal angulation of 21.5–25.1° (95% CI) and sagittal angulation 61.3–66.1° (95% CI).

For the supra-acetabular corridor, all sections exhibit an S-shaped configuration with proximal and distal expansion and mid-corridor constriction. The longest anatomical axis, located from the most anterior point of the AIIS to the midpoint of the transverse diameter of the PSIS (section A2), measured 116.7–125.3 mm (95% CI). The medial-lateral cortical spacing at the mid-corridor constriction of section A2 was 12.3–13.5 mm (95% CI), and the distance from the most anterior point of the AIIS to the mid-corridor constriction was 47.8–51.5 mm (95% CI). Screw S3 demonstrated horizontal and sagittal angles of 58.2—61.3° and 23.0—25.6°, respectively (both 95% CI).

No statistically significant differences in anthropometric measurements (height or weight) were observed between male and female cohorts. Comprehensive results for each parameter are systematically presented in Table 1.

## 4 Discussion

This study employed a dataset comprising high—resolution pelvic computed tomography (CT) scans obtained from a cohort of normally developed adolescents aged 11 to 14 years for three—dimensional modeling and anatomical analysis, with a specific focus on exploring osseous anatomical corridors within the adolescent pelvis that are appropriate for the insertion of Schanz screws. The results demonstrate that, in the studied adolescent population, osseous corridors in the iliac wing and supra—acetabular region of the pelvis are suitable for Schanz screw implantation. Through the observation of the anatomical morphology and the measurement of the geometric dimensions of these osseous corridors via virtual visualization planes, the anatomical boundaries of the optimal implantation corridors for Schanz screws used in external fixators can be precisely defined.

Pelvic anatomical research has promoted the application of minimally invasive surgical techniques in the treatment of pelvic fractures (14–17). Studies have found that there are natural osseous corridors available for screw placement in the adult pelvic ring, around the acetabulum, and at the sacroiliac joint (18–22). Minimally invasive techniques, which utilize three—dimensional reduction assisted by traction (6), allow for the percutaneous insertion of screws or external fixator Schanz screws through the osseous anatomical corridors of the pelvis (2, 23). Consequently, this has altered the traditional open reduction and internal fixation (ORIF) approach (24), which previously required complex surgical incisions and extensive soft tissue dissection to achieve fracture reduction and fixation. As a result, MIS reduces surgical trauma and complications while accelerating the rehabilitation process (3, 25, 26). Unfortunately, the immature pediatric pelvis, which contains growth plates, has smaller three-dimensional skeletal dimensions than those of adults, undergoes rapid developmental changes, and exhibits significant individual variations. To date, research on pediatric pelvic anatomical bone corridors has been limited. Consequently, these factors have restricted the widespread adoption of minimally invasive techniques in the treatment of pediatric pelvic fractures. Literature shows that, due to narrower surgical corridors and weaker bones, traditional fluoroscopy—guided screw placement risks cortical breach, epiphyseal injury, unstable fixation, and increased radiation exposure (7, 12). Due to the presence of the triradiate cartilage and the limited skeletal geometric space, this study focuses on the anatomical analysis of the osseous corridors in the pediatric pelvis above the iliac wing and acetabulum for the placement of external—fixation Schanz screws, aiming to promote the application of minimally invasive surgery (MIS) techniques in the treatment of pediatric pelvic fractures.

This study employed virtual visualization planes to analyze the anatomical bone corridors of the adolescent pelvis. Anatomical landmarks on the pelvis were selected to determine the entry points for screw insertion. Measuring cortical bone distances and corridor lengths in bony corridors defines their anatomical boundaries, providing anatomical evidence for selecting appropriate screw diameter, optimal insertion depth, and suitable angle. This study's methodology aligns with preoperative simulation and planning in digital orthopedics. These approaches have shown great potential for widespread application and remarkable effectiveness in treating adult pelvic and acetabular fractures. For instance, Lang's team (27) crafted 3D—printed guides to precisely guide the Schanz screw placement during surgery, ensuring efficient execution of preoperative plans, substantially enhancing fixation stability, and effectively reducing screw—placement—related complication risks. Computer-assisted preoperative surgical planning is particularly well-suited for adolescents in their developmental stages, as it enables the formulation of individualized preoperative plans tailored to patients at different developmental stages, thereby playing a crucial role in enhancing surgical safety and clinical outcomes. When integrated with navigation and robot-assisted techniques, it allows for even more minimally invasive and precise screw placement. Clinicians can refer to the methodology of this study to preoperatively determine the three-dimensional anatomical parameters of the optimal screw insertion corridor, and intraoperatively insert a screw of appropriate diameter to the predetermined depth based on the predefined entry point and angle. Similar to spinal pedicle screw placement, once the entry point and angles are clearly determined, the screw can be smoothly inserted into the narrow bony corridor, thereby shortening operative time, reducing complications, and ensuring satisfactory surgical outcomes.

The iliac wing corridor is a bony protuberance in the anterior iliac wing of the pelvis and serves as one of the commonly used bony corridors. Öztürk (5) analyzed CT data from 400 adult pelvises and confirmed the existence of a bony corridor running from the iliac crest to the lesser sciatic notch, through which dual-column screws can be inserted. Sun (28) et al. further demonstrated that by conducting an anatomical analysis of the safe corridor, and optimizing the screw insertion pathway, screws with larger diameters could be used, thereby increasing the fixation strength and fracture stability. In this study, given the smaller pelvic dimensions in adolescents and the presence of unclosed triradiate cartilages, the study selected 4.0 mm Schanz screws for simulated insertion, with the plane above the acetabulum chosen as the endpoint for screw insertion depth. When the Schanz screws were inserted parallel to the planes of the anterior and posterior boundaries of the corridor, the screw spacing was maintained at approximately 35 mm, which could meet the stability requirements for dual-screw fixation. If larger screws are to be used, adjustments are necessary: the anterior insertion point must be moved posteriorly, and the posterior insertion point must be moved anteriorly, to accommodate the iliac wing corridor's anatomical constraints. From another perspective, the cross—section of the iliac wing corridor presents an hourglass shape, with a crucial narrow area located around 30 millimeters below the surface of the iliac crest. This indicates that if the screw is inserted off the axis of the bony corridor, it is highly likely to penetrate the cortical bone at this narrow area, thereby adversely affecting the fixation strength. In the study, the angles between the S1 and S2 screws and the trunk's horizontal and sagittal planes precisely capture the corridor's spatial orientation, offering crucial guidance for screw insertion direction.

The supra—acetabular corridor, which extends from the anterior inferior iliac spine (AIIS) directly to the posterior superior iliac spine (PSIS), is a bony passage. Compared with the iliac wing corridor, the supra—acetabular corridor not only boasts a wider spatial scope but also has a greater length. These significant advantages make it more appealing in the field of clinical applications. This study reveals that the cross—section of this corridor presents a unique S—shape with a relatively spacious internal space. Measurement data from the study population indicate that the narrowest transverse diameter of the middle plane of the corridor ranges from 12.3 to 13.5 millimeters (95% confidence interval). This width is sufficient to ensure the smooth insertion of Schanz pins or commonly used clinical screws. This result is consistent with the conclusions from adult imaging studies. Bondt (29) et al. believe that the supra—acetabular corridor has ample space and can accommodate screws with a diameter of up to 7.3 millimeters. Ruewald (30) et al.'s research points out that screws not exceeding a length of 97 mm in females and 106.4 mm in males were, in 95% of the evaluated cases, insertable without cortical bone penetration. This is relatively close to the result of the longest trajectory (116.7–125.3 mm; 95% CI) aligned with the AIIS-PSIS central plane obtained in this study. Based on the findings of this study, when inserting screws, they should be oriented towards the posterior superior iliac spine, and the insertion depth should be adjusted flexibly according to the actual fixation requirements. It is particularly important to note that, according to Morandi's research (31), the longer the length of the inserted screw, the smaller its safe insertion range and the higher the risk of penetrating the cortical bone. The anterior inferior iliac spine has a superficial location and a limited range, which greatly facilitates the precise localization of the screw entry point during surgery. Lidder (32) et al.'s research suggests that the entry point should be kept away from the acetabulum as much as possible to reduce the risk of the screw entering the joint capsule. For adolescents who have not yet fully developed, this study holds that screws should be avoided from being inserted too close to the superior margin of the acetabulum to prevent damage to the epiphyseal plate and the labrum—cartilage complex, as such damage may disrupt the normal blood supply to the acetabulum and subsequently have an adverse impact on its development.

This study had several limitations. First, the single-center design as well as a homogeneous ethnic group and a small sample size (*n* = 34) introduce potential selection bias; however, the narrow 95% confidence intervals observed across all measured parameters suggest robust statistical trends, thereby supporting the clinical applicability of these findings to similar demographic cohorts. No formal power analysis (neither *a priori* nor post-hoc sensitivity) was performed due to the exploratory nature of this study and the absence of prior data in the 11–14 year age group. While the narrow CIs support descriptive precision, the study was underpowered to detect small-to-moderate effect sizes (e.g., small sex-related differences). Therefore, non-significant comparisons should be interpreted cautiously. Second, although the study did not specifically analyze gender-based differences, the balanced gender distribution (1:1 ratio) and lack of significant anthropometric variations (e.g., height, weight) suggest minimal confounding effects from this variable. Third, the age range was restricted to adolescents aged 11–14 years, thereby excluding prepubertal children and postpubertal adolescents. Additionally, this study simulated screw placement using 4 mm Schanz screws, and the corridor measurements may vary with different screw diameters. Future studies should validate these results across a range of screw diameters to optimize clinical decision-making.

While the current study provides initial anatomical evidence for CT-based preoperative planning in this age group, prospective validation through multi-center trials involving larger and more diverse patient populations remains critically important to confirm its generalizability. Bone age-stratified analyses would further elucidate developmental variations in pelvic anatomy pediatric age groups, as skeletal maturation significantly influences optimal screw trajectory parameters. These findings collectively advance precision surgery in pediatric pelvic fractures by enabling more accurate screw placement, reduced surgical trauma, shorter operative times, and decreased radiation exposure—ultimately improving clinical outcomes while minimizing complications.

## 5 Conclusions

The findings of this study reveal that, among the population studied, both the iliac wing corridor and the supra—acetabular corridor of the pelvis offer feasible bony routes for the insertion of Schanz screws. However, given the three—dimensional spatial constraints of the adolescent pelvis and the anatomical boundaries of these bony corridors, meticulous preoperative planning is of utmost importance. This planning should precisely determine the screw entry point and its trajectory, thus reducing the risk of perforation and enhancing the stability of fixation to the greatest extent possible. This work establishes a methodological foundation for integrating advanced technologies such as navigation systems and robotic assistance into pediatric orthopedic practice, particularly within the specialized domain of orthopedic trauma care.

## Data Availability

The original contributions presented in the study are included in the article/Supplementary Material, further inquiries can be directed to the corresponding author/s.
